# Pathological calcification in canine tendon-derived cells is modulated by extracellular ATP

**DOI:** 10.1007/s11259-024-10331-1

**Published:** 2024-02-21

**Authors:** Danae E. Zamboulis, Neil Marr, Alaa Moustafa, Richard Meeson, Isabel R. Orriss, Chavaunne T. Thorpe

**Affiliations:** 1 Department of Comparative Biomedical Sciences, Royal Veterinary College, Camden, London, UK; 2 Department of Clinical Sciences, School of Veterinary Medicine, Aristotle University of Thessaloniki, 54124 Thessaolinki, Greece; 3 Department of Clinical Science and Services, Royal Veterinary College, London, UK; 4 Department of Surgery, Anaesthesiology, and Radiology, Faculty of Veterinary Medicine, Kafr Elshiekh University, Kafr Elshiekh, Egypt

**Keywords:** Tendon, Calcification, Canine, Mineralisation, ATP

## Abstract

Tendon calcification is a commonly associated with degenerative tendinopathy of the Achilles tendons in dogs. It is characterised by the formation of calcific deposits and is refractory to treatment, often re-forming after surgical removal. Little is known about its pathogenesis and therefore the aims of this study were to develop an in vitro model of canine tendon calcification and use this model to investigate mechanisms driving calcification. Cells from the canine Achilles tendon were cultured with different calcifying media to establish which conditions were best able to induce specific, cell-mediated calcification. Once optimum calcification conditions had been established, the effect of ATP treatment on calcification was assessed. Results revealed that 2 mM di-sodium phosphate combined with 2 mM calcium chloride provided the optimum calcifying conditions, increasing calcium deposition and expression of osteogenic-related genes similar to those observed in tendon calcification in vivo. ATP treatment inhibited calcification in a dose-dependent manner, reducing calcium deposition and increasing cell viability, while osteogenic-related genes were no longer upregulated. In conclusion, the in vitro model of canine tendon calcification developed in this study provides the ability to study mechanisms driving tendon calcification, demonstrating that ATP plays a role in modulating tendon calcification that should be explored further in future studies.

**Supplementary Information:**

The online version contains supplementary material available at 10.1007/s11259-024-10331-1.

## Introduction

Tendon calcification is characterized by formation of calcific deposits within tendon, and is commonly associated with tendon pathology (Darrieutort-Laffite et al. 2018). Calcification has been reported in 30% of dogs presenting with shoulder pain, and has been detected in 13% and 20% of dogs presenting with supraspinatus and Achilles degeneration respectively (Maddox et al. 2013; Canapp et al. 2016; Gamble et al. 2017). Tendon calcification is also common in humans, likewise most often occurring in the supraspinatus and Achilles tendons in middle-aged individuals (Sansone et al. 2018; Giai Via et al. 2020). In humans, it is thought that calcium deposition within tendon is an active process, mediated by the resident cells (Uhthoff 1975; Darrieutort-Laffite et al. 2018). In canine tendons, hypoechoic areas surround regions of calcification, indicating the presence of inflammation (Mistieri et al. 2012). However, little is known regarding the pathogenesis of the disease in either species, and treatment options remain limited, often resulting in prolonged symptoms which do not resolve. Indeed, studies have shown that, while initial outcome after surgery for calcification of the supraspinatus tendons is good, long-term re-evaluation revealed reformation of calcification in all dogs assessed (Laitinen and Flo 2000, Lafuente et al. 2009).

A small number of studies have investigated the response of tendon-derived cells to calcifying media in vitro (Yue et al. 2016; Chen et al. 2019; Darrieutort-Laffite et al. 2019), however these studies have used levels of phosphate that far exceed those seen under physiological and pathological conditions in vivo (in excess of 5 mM). This results in dystrophic, non-specific deposition of mineral rather than specific cell-mediated calcification (Perpétuo et al. 2019). Thus, no in vitro models to induce specific tendon-derived cell calcification currently exist. Nevertheless, studies in other tissues have shown that with the appropriate conditions it is possible to model cell-mediated calcification in vitro*.* For example, in osteoblasts, the bone-forming cells, physiological mineralisation can be induced by culturing with 2–5 mM β-glycerophosphate (Taylor et al. 2014; Perpétuo et al. 2019). Whereas vascular calcification, another form of pathological calcification, can be modelled using vascular smooth muscle cells (VSMCs) cultured with sodium phosphate (≤ 5 mM) with or without calcium (usually as calcium chloride, ≤ 3 mM) (Patel et al. 2019).

Extracellular adenosine triphosphate (ATP) is an established signalling molecule that can act via P2 receptors to induce a range of functional effects in many different cell types (Burnstock 2007). ATP and related compound uridine triphosphate (UTP) have been shown to be potent inhibitors of physiological bone mineralisation, acting via both P2 receptor dependent and independent pathways (Hoebertz et al. 2000; Orriss et al. 2007, 2012). Similarly, ATP and UTP are also able to inhibit vascular calcification albeit via different mechanisms (Villa-Bellosta and Sorribas 2013, Patel et al. 2018). Whilst some of the actions of ATP and UTP are via P2 receptor mediated signalling, these compounds are also hydrolysed by ecto-nucleotide pyrophosphatase/phosphodiesterase 1 (NPP1) to produce pyrophosphate (PP_i_). Since PP_i_ is a ubiquitous and potent mineralisation inhibitor, some of the actions of ATP and UTP on mineralisation/calcification processes are likely to be indirect (Fleisch and Bisaz 1962, Orriss 2020).

The first aim of this study was to develop a novel, in vitro model of tendon calcification. The second aim was to use this model to investigate mechanisms driving calcification in cells derived from the canine Achilles tendon.

## Methods

### Isolation and culture of tendon-derived cells

Skeletally mature dogs (*n* = 3) euthanised for reasons unrelated to orthopaedic disease and donated to the cadaveric tissue donation programme with ethical (URN 2021 2072–2) and owner consent provided the samples (Donor details are displayed in Table 1). Within 3 h of euthanasia, the caudal hock region was aseptically prepared and through a linear skin incision, a 1.5 cm section of distal Common Calcaneal tendon (Gastrocnemius and conjoined tendon components) was isolated and resected from both hindlimbs. No tendons displayed macroscopic signs of injury or disease and samples were stored in Dulbecco’s Modified Eagle Media (DMEM) with primocin (100 µg/ml) prior to digestion at 5 °C for up to 24 h. The epitenon was then removed and tendons were finely minced and digested with 1 mg/mL pronase E (39052, VWR) per 1 g tissue for 2–4 h at 37 °C under constant agitation. Following pronase digestion, tissue was digested for a further 16 h with 0.5 mg/ml collagenase type IV (CLS-4, Lorne Laboratories) and 1 mg/mL dispase II (17105041, Invitrogen) at 37 °C with constant agitation. Cell suspensions were strained through a 70 µm filter and plated in T-75 cell culture plates. Tendon derived cells (TDCs) were cultured in low glucose DMEM supplemented with 10% FBS and antibiotic–antimycotic (complete mixture abbreviated to DMEM) until 80% confluence was reached, with media changes every 3–4 days.

**Table 1:** Details of donors from which Achilles tendons were harvested

Donor	Age	Sex	Breed
2	1 year 4 months	Male entire	Cocker spaniel
3	2 year 2 months	Male neutered	French bulldog
4	7 years	Female	Golden retriever

### Establishing optimum calcification conditions for tendon-derived cells

**Fig. 1:**
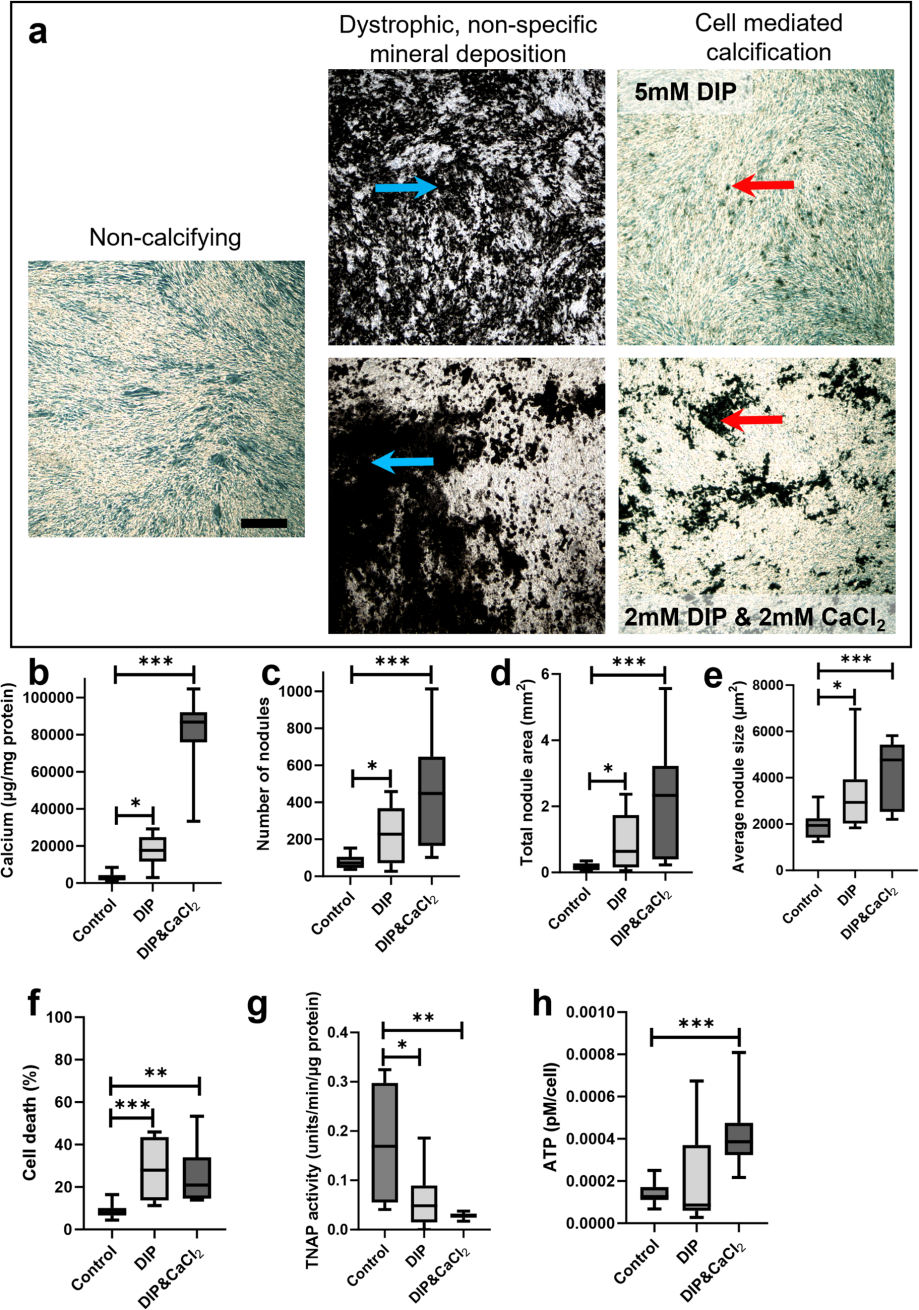
**Developing a model of calcification in tendon-derived cells.** (**a**) Phase contrast images of non-calcifying cells, dystrophic mineral deposition and cell mediated calcification. Non-specific dystrophic calcification occurs when phosphate and/or calcium is used to excess and is illustrated by the blue arrows. Cell-mediated calcification is less widespread and is shown by the red arrows. 2 mM DIP & 2 mM CaCl_2_ induced more cell-mediated calcification than 5 mM DIP. Scale bar: 100 µm. **(b)** Both calcifying conditions induced calcium deposition with a greater increase seen with DIP & CaCl_2_ (measured via colourimetric assay). Image analysis of fixed cell layers shows the effect of different calcifying conditions on **(c)** number of calcification nodules, **(d)** total nodule area and **(e)** average nodule size. Culture in calcifying conditions is associated with **(f)** increased cell death, **(g)** decreased tissue non-specific alkaline phosphatase (TNAP) activity and **(h)** higher ATP release. *p < 0.05; **p < 0.01; ***p < 0.001

TDCs were cultured in phenol red-free DMEM supplemented with a range of phosphate sources that are commonly used to induce calcification. These included β-glycerophosphate and sodium phosphate dibasic (Na_2_HPO_4_, abbreviated to DIP) alone or in combination with calcium chloride (CaCl_2_). Based on pilot experiments (Fig. 1), it was determined that two types of calcifying media would be taken forward for detailed study; 5 mM DIP and 2 mM DIP & 2 mM CaCl_2_. Cells from each donor were seeded in 24-well plates at a density of 25,000 cells per well. Control cells were cultured under non-calcifying condition in DMEM supplemented with 50 μg/ml ascorbic acid. Calcifying cells were grown in DMEM supplemented with 50 μg/ml ascorbic acid and, either 5 mM DIP or 2 mM DIP & 2 mM CaCl_2_. To determine the effects of ATP on calcification in tendon-derived cells, both non-calcifying and calcifying media were supplemented with 10–100 µM ATP for the duration of the experiment. For all cultures, half media changes were performed every 3–4 days, and experiments were terminated after 8 days.

### Assessment of calcium deposition

The amount of calcium deposited by cells in each treatment group was quantified in two ways; image analysis and colorimetric assay. For image analysis, media was removed, wells were washed with PBS and fixed with 2.5% glutaraldehyde for 5 min, followed by 2 × PBS washes. Cells were rinsed 3 times with 70% ethanol and left to air dry at room temperature. Plates were scanned at 800 dpi (Epson Perfection 4990 Photo) and nodule number, total nodule area and average nodule size were measured using ImageJ as previously described (Perpétuo et al. 2019). In addition, plates were imaged using phase-contrast microscopy to visualise the calcification (Axiovert 35TV inverted microscope (Carl Zeiss Ltd, Cambridge, UK) with a 20 × LD ACHROPLAN objective.

To measure the calcium deposition by colourimetric assay, monolayers were washed twice with PBS and incubated overnight at room temperature with 0.6 M HCl. Calcium content was determined by measuring the stable interaction with *o*-cresolphthalein using a commercially available kit (Sigma-Aldrich, UK) and corrected for total protein concentration using the Bradford assay (Sigma-Aldrich, UK) (Patel et al. 2018).

### Cell viability assay

Cells were cultured for 8 days in non-calcifying or calcifying media, with or without ATP. Cell viability was assessed using a CytoTox 96 Non-radioactive cytotoxicity assay (Promega) as described previously (Orriss et al. 2012). Briefly, media was removed from cultured cells and replaced with serum-free media. Cells were incubated at 37 °C in serum free media for 45 min to 1 h and media (25 µl) were collected to determine medium lactate dehydrogenase (LDH) levels (viability). To determine total cellular LDH levels, cells were lysed by addition of 1% Triton X-100 (v/v) to the media, and samples collected after 20 min. Assays were conducted according to the manufacturer’s instructions and samples were incubated at room temperature before reading at 495 nm. Cell viability (shown as percentage of dead cells) was calculated by expressing medium LDH as a percentage of the total cellular LDH.

### Determination of tissue non-specific alkaline phosphatase (TNAP) activity

Cells were cultured as described above for 8 days. TNAP activity was assessed in cell lysates using a colorimetric assay (Sigma Aldrich), as previously described (Orriss et al. 2012). Enzyme activity was normalised to total protein content measured using the Bradford assay.

### Measurement of ATP release

Release of ATP from non-calcifying and calcifying TDCs was measured after 8 days in culture. To avoid confounding effects of any treatments on the ATP measurements, culture medium was removed, cell layers washed and cells incubated with serum-free DMEM for 1 h. Following sample collection, ATP levels were measured immediately (Orriss et al. 2009). ATP release was normalised to cell number and viability monitored using the assay described above.

### Gene arrays for tendon and osteogenic-related genes

To establish the effect of calcifying media and ATP treatment on gene expression, custom gene arrays for a panel of 36 tendon- and osteogenesis-related genes were performed (RT2 Profiler PCR Array, Qiagen). The list of genes measured is available in supplementary Table 1. Based on previous results, the following groups were compared: non-calcifying media; non-calcifying media plus 100 µM ATP; 2 mM DIP & 2 mM CaCl_2_; 2 mM DIP & 2 mM CaCl_2_ plus 100 µM ATP. After 8 days of culture, media was removed from cells and monolayers washed with PBS. Cells were lysed with Trizol and samples frozen prior to gene expression analysis. Total mRNA was extracted using Direct-zol RNA Microprep Kits (Zymo Research) with the inclusion of a DNase step and RNA quality assessed by measuring 260/280 nm absorbance ratio. 0.5 µg of mRNA was reverse transcribed to cDNA using an RT2 First Strand Kit (Qiagen) and arrays were performed according to the manufacturer’s instructions (RT2 Profiler PCR arrays, Qiagen). Gene expression was normalised using a panel of 6 housekeeping genes, and relative expression calculated according to the 2^−ΔCT^ method (Livak and Schmittgen 2001). Statistical analysis was performed using the GeneGlobe data analysis tool (Qiagen).

### Statistical analysis

Data were tested for normality using the Kolmogorov–Smirnov test. Data did not follow a normal distribution therefore Kruskal–Wallis tests followed by post-hoc analysis using the two-stage linear step-up procedure of Benjamini et al (2006) were used to determine significance between experimental groups. Significance was set at *p* < 0.05, with a trend towards significance at *p* < 0.1. Data analysis and visualisation was performed in GraphPad Prism (v10.0.2). Box plots show mean and interquartile range, with whiskers representing maximum and minimum data points.

## Results

### Developing a model of calcification in tendon-derived cells

Treatment of TDCs with both calcifying media resulted in specific, cell-mediated calcification rather than dystrophic, non-specific mineral deposition. There was significant increase in calcium deposition in calcifying cells compared to non-calcifying conditions, and a greater increase seen in response to DIP & CaCl_2_ treatment (Fig. 1a & b). Image analysis of fixed cell layers revealed that culturing cells in the presence of 5 mM DIP or 2 mM DIP & 2 mM CaCl_2_ resulted in a significant increase in the number of nodules formed (Fig. 1c). Similarly, total nodule area and average nodule size increased in response to calcifying media, with a greater response seen in cells treated with both DIP and CaCl_2_ (Fig. 1d & e). Cell viability was relatively high in cells cultured in non-calcifying media, with less than 10% cell death on average. Treatment with both calcifying media resulted in a significant increase (~ threefold) in the percentage of dead cells (Fig. 1f). TNAP activity was low in all groups with a significant reduction observed when cells were treated with calcifying media (Fig. 1g). Culturing cells with DIP only did not affect ATP release, but treatment with DIP and CaCl_2_ resulted in a significant increase in ATP release (Fig. 1h).

### Effect of ATP treatment on calcification

**Fig. 2:**
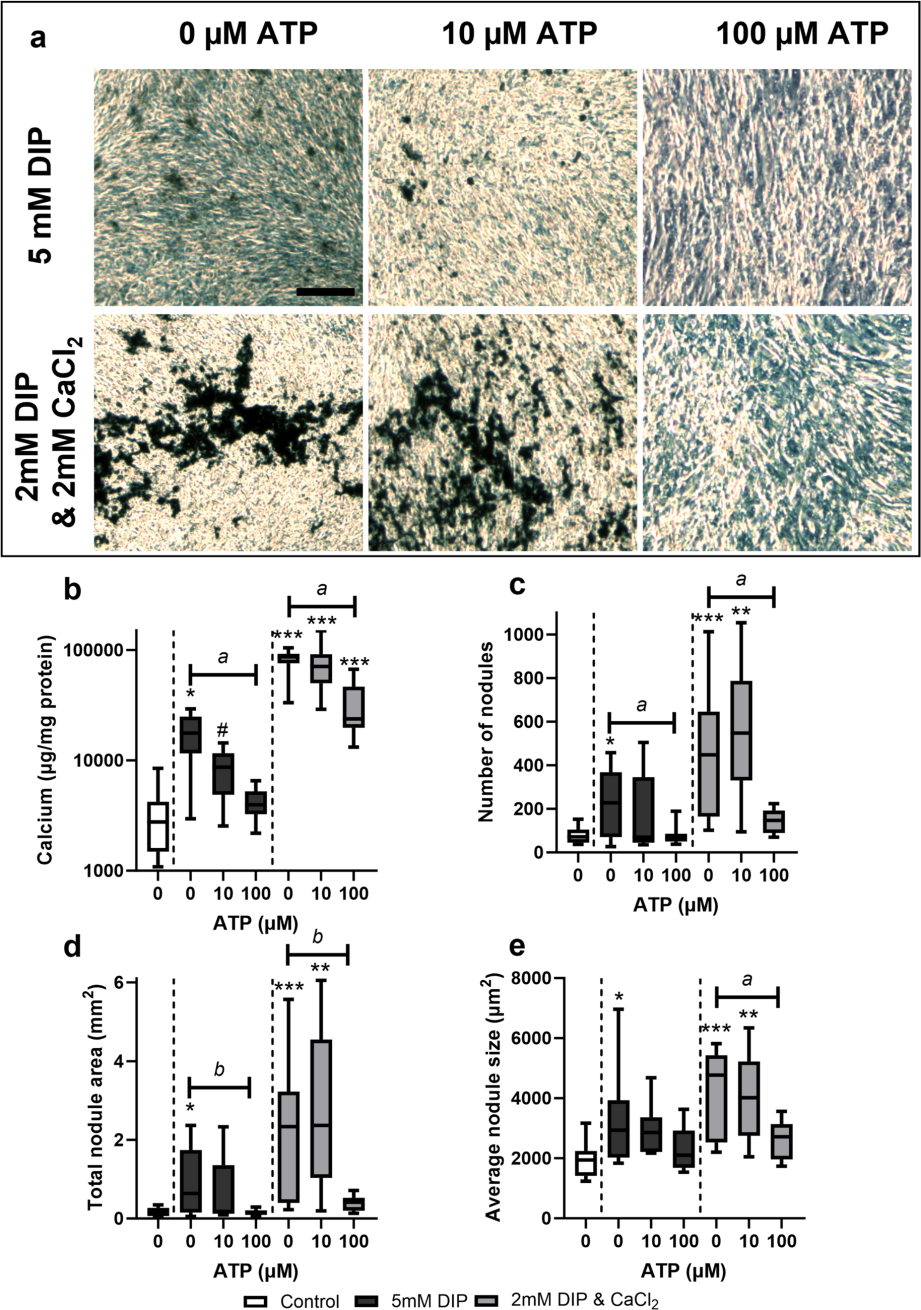
**Effect of ATP treatment on calcium deposition under different calcifying conditions.** (**a**) Representative phase contrast images demonstrate that ATP treatment reduces calcification in both calcium conditions tested. **(b)** the amount of calcium deposited decreased with increasing ATP concentration but remained significantly higher in cells treated with DIP and CaCl_2_. Image analysis of fixed cell layers showed that treatment with 100 µM ATP decreased nodule number (**c**), total nodule area (**d**), and average nodule size (**e**). Significant differences are shown relative to non-calcifying controls (# *p* < 0.1; *p < 0.05; **p < 0.01; ***p < 0.001) or to ATP concentration (^a^*p* < 0.1; ^b^*p* < 0.05)

Treatment of calcifying TDCs with ATP at a concentration of 100 µM caused a reduction in calcium deposition, particularly evident in cells cultured with 2 mM DIP & 2 mM CaCl_2_ (Fig. 2a). The amount of calcium deposited, as measured by colourimetric assay, was not affected by treatment with 10 µM ATP, whereas 100 µM ATP reduced calcium levels in calcifying conditions although these remained higher than in non-calcifying groups (Fig. 2b). Image analysis of fixed cell layers revealed that treatment with 10 µM ATP had no effect on the number of nodules formed, whereas 100 µM ATP reduced number of nodules to levels similar to those in cells cultured in non-calcifying media. Similar results were seen when measuring total and average nodule area, with a greater effect seen in cells cultured with 2 mM DIP & 2 mM CaCl_2_ compared with 5 mM DIP in the presence and absence of ATP (Fig. 2c-e). ATP treatment had no effect on calcification in non-calcifying TDCs (supplementary Fig. 1).

### Effect of ATP treatment on cell viability and tissue non-specific alkaline phosphatase (TNAP) activity

**Fig. 3:**
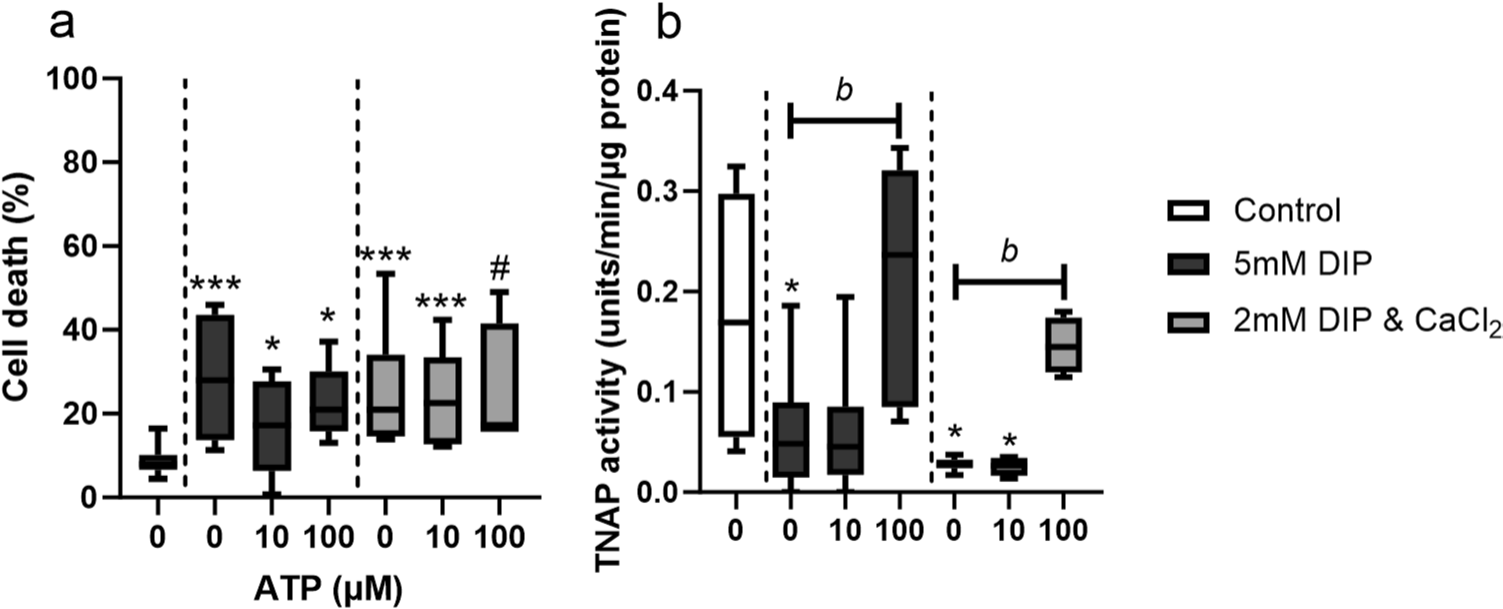
**Effect of ATP treatment on cell viability and tissue non-specific alkaline phosphatase (TNAP) activity under different calcifying conditions.** Percent cell death (**a**) tended to decrease with increasing ATP concentrations, but remained higher than under non-calcifying conditions. The decrease in TNAP activity (**b**) seen under calcifying conditions was reversed by treatment with ATP at a concentration of 100 µM only. Significant differences are shown relative to non-calcifying controls (# *p* < 0.1; *p < 0.05; **p < 0.01; ***p < 0.001) or to ATP concentration (^b^*p* < 0.05)

The reduction in cell viability that occurred when cells were cultured in calcifying media was partially reversed with the addition of ATP at 100 µM, although the percentage of cell death remained significantly greater than in non-calcifying controls (Fig. 3a). Treatment with 10 µM ATP did not affect TNAP activity, whereas 100 µM ATP returned TNAP activity to levels similar to those seen in non-calcifying conditions (Fig. 3b). ATP treatment had no effect on cell viability or TNAP activity in non-calcifying TDCs (supplementary Fig. 1).

### Effect of ATP treatment on ATP release

**Fig. 4:**
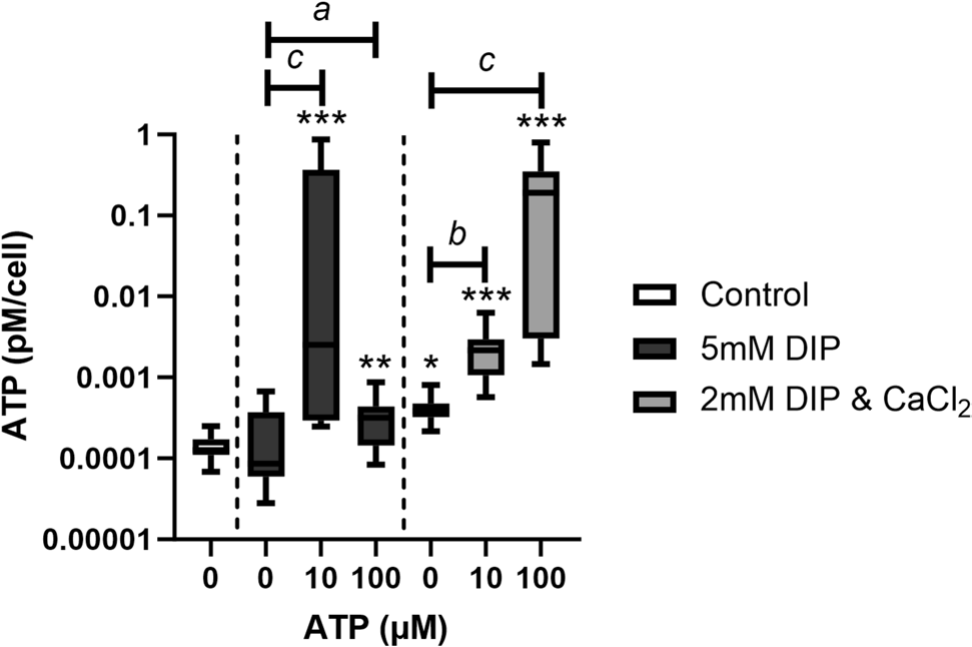
**Effect of ATP treatment on ATP release under different calcifying conditions.** Treatment with 10 µM and 100 µM ATP caused an increase in ATP release under calcifying conditions. Significant differences are shown relative to non-calcifying controls (*p < 0.05; **p < 0.01; ***p < 0.001) or to ATP concentration (^a^*p* < 0.1; ^b^*p* < 0.05; ^c^*p* < 0.01)

Treatment of cells under calcifying conditions with ATP significantly increased ATP release compared to non-calcifying controls. (Fig. 4). In cells treated with 5 mM DIP, this effect was more pronounced when 10 µM ATP was used, whereas in cells treated with 2 mM DIP & 2 mM CaCl_2_, treatment with 100 µM ATP had the largest effect. ATP treatment had no effect on ATP release in non-calcifying TDCs (supplementary Fig. 1).

### Effect of calcifying media and ATP treatment on gene expression

**Fig. 5:**
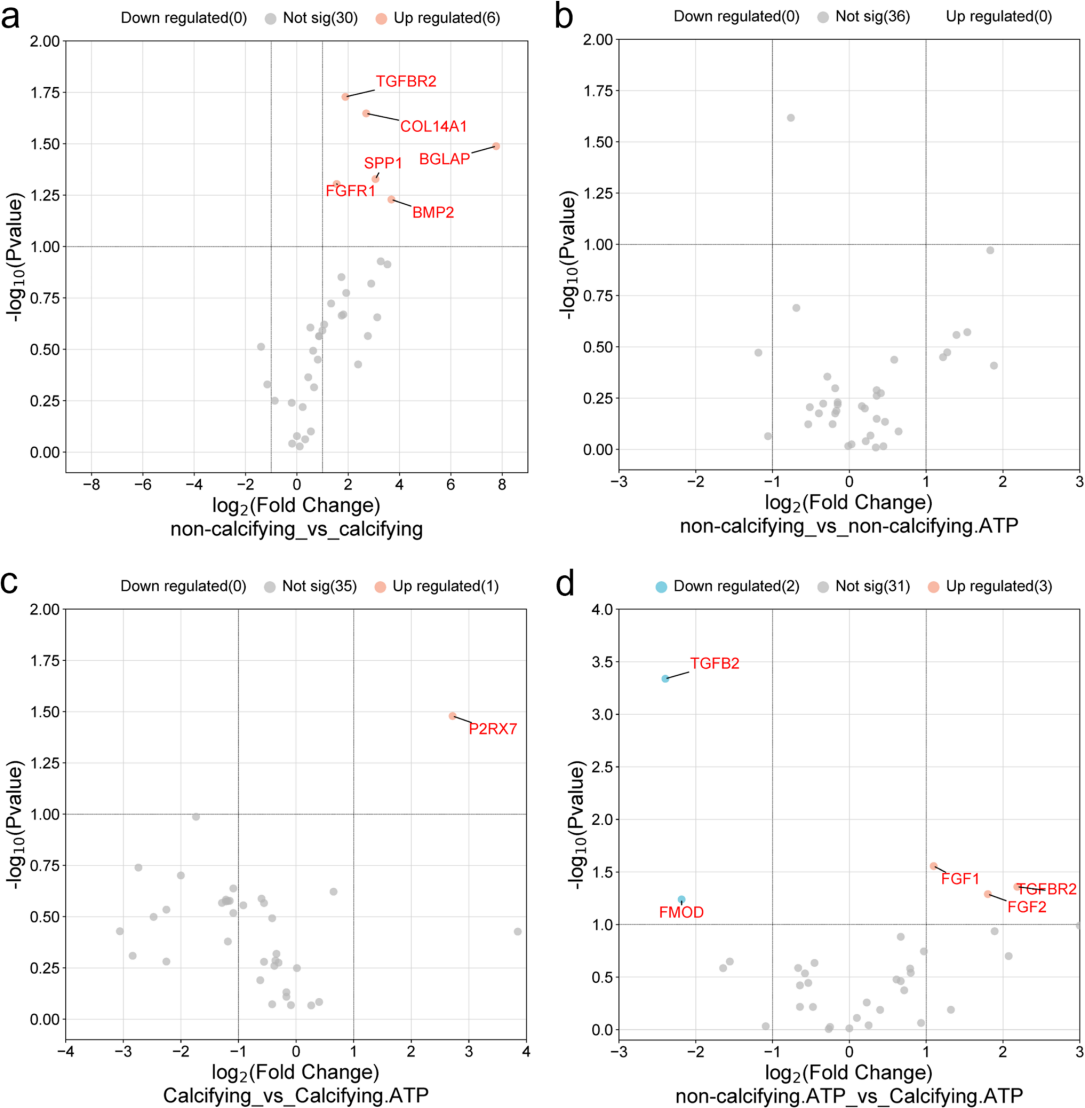
**Effect of calcifying media and ATP treatment on expression of tenogenic and osteogenic related genes.** Volcano plots showing gene expression in non-calcifying cells compared to calcifying cells (**a**) non-calcifying compared to non-calcifying, ATP-treated cells (**b**) calcifying compared to calcifying, ATP-treated cells (**c**) non-calcifying, ATP-treated cells compared to calcifying, ATP-treated cells (**d**). Significantly (*p* < 0.1, fold change > 2) regulated genes are labelled, red are upregulated, blue are down regulated

Several genes were upregulated in calcifying TDCs compared to controls, including the osteogenic markers *SPP1* (osteopontin) and *BGLAP* (osteocalcin) and those coding cell signalling proteins (*FGFR1, BMP2, TGFBR1*) (Fig. 5a). However, there were few changes in the expression of tendon-related genes. There were no differences in gene expression in non-calcifying cells with and without ATP treatment, indicating that ATP treatment alone had little effect on cell phenotype (Fig. 5b). In calcifying cells, addition of ATP caused an upregulation of the P2X7 receptor gene only (Fig. 5c). When comparing control and calcifying cells both treated with ATP, growth-factor related genes were upregulated (*FGF1, FGF2, TGFBR2*), and *TFGBR2* and *FMOD* were downregulated in the calcifying group (Fig. 5d).

## Discussion

We have developed an in vitro model in which specific, cell-mediated calcification can be induced in canine Achilles tendon-derived cells, without dystrophic mineral deposition or excessive cell death. We have used this model to demonstrate that tendon cell mineralisation can be inhibited by addition of ATP, supporting our hypothesis that dysregulation of pathways that regulate calcification could contribute to development of pathological tendon calcification. Further, we have identified key genes that are regulated both by calcification and ATP, providing targets for future studies to unpick the mechanisms governing tendon calcification, and ultimately for therapy.

There are several limitations associated with this study, including variations in donor breed, age and sex which may have contributed to the large variability in the data between individuals. Despite this, we were able to identify clear differences between calcifying and non-calcifying conditions and with ATP treatment. Another consideration is the use of cells from normal rather than diseased tendons, in which the pathways driving mineralisation may differ, and therefore this should be a focus of future studies. The sensitivity of the methods used to measure calcium deposition should also be considered. The image analysis methods used in this current study were developed to quantify physiological in vitro bone formation; however, the calcification nodules formed by TDCs are relatively small compared to bone nodules formed by osteoblasts (Perpétuo et al. 2019). Thus, it is possible that some of the smaller deposits could have been missed during the analysis. Therefore, we also utilised a colorimetric assay to measure deposited calcium. This approach, which is widely used in the study of vascular calcification, appeared to be more sensitive as it detected changes in the level of calcium deposition with 10 μM ATP as opposed to the 100 μM ATP with image analysis. Despite the differences in sensitivity, both methods showed the same overall trends.

A small number of studies have investigated calcification in tendon-derived cells, demonstrating that in rat tendon-derived stem cells, mineralisation can be inhibited by magnesium, with this inhibitory effect reversed by high concentrations of ATP (above 100 μM) (Yue et al. 2016). Similarly, stem/progenitor cells derived from mouse tail tendon show increased mineralisation when treated with interleukin-1β in the presence of osteogenic media (Chen et al. 2019). Cells derived from the rotator cuff tendon have also been reported to mineralise when treated with osteogenic media, which was accompanied by an increase in TNAP activity (Darrieutort-Laffite et al. 2019). However, these studies used high (10 mM) concentrations of β-glycerophosphate (BGP) to induce calcification, which can cause widespread, non-specific mineral deposition distinct from true bone formation or cell-mediated calcification, and also reduces cell viability and TNAP expression (Orriss et al. 2007; Hortells et al. 2015; Perpétuo et al. 2019). The results of these studies should be interpreted with caution, as they are unlikely to recapitulate the physiological environment that cells experience in vivo.

In the current study, we studied the effect of two types of calcifying media on tendon cell calcification. While both were able to induce specific, cell-mediated calcification, a greater degree of calcification occurred in cells treated with a combination of DIP and CaCl_2_. The reasons for this are unclear, but it could suggest that any phenotypic changes in TDCs that cause calcification require changes in extracellular calcium and phosphate (as opposed to just phosphate). Further work is clearly required to understand how changes in the extracellular environment drive pathological tendon calcification.

TNAP is a key enzyme involved in promoting physiological bone mineralisation. It hydrolyses a range of phosphate-containing molecules, including the inhibitor PP_i_, to produce the phosphate needed to drive mineralisation. Increased expression of TNAP in soft tissues has been associated with driving the development of pathological calcification including arterial medial calcification (Sheen et al. 2015). Therefore, this study investigated whether TNAP could play a role in calcification in tendon-derived cells. While TNAP activity was reduced in response to calcifying media, the absolute values of TNAP activity are approximately 1000-fold less than in mature osteoblasts that are actively mineralising (Patel et al. 2019). Therefore, TNAP is unlikely to play a significant role in driving mineral deposition in TDCs. However, TNAP activity was responsive to ATP treatment, returning to levels seen in non-calcifying controls.

ATP dose-dependently inhibited calcification, consistent with the inhibitory effect reported on bone mineralisation and vascular calcification (Hoebertz et al. 2000, Orriss et al. 2007, Orriss et al. 2012, Villa-Bellosta and Sorribas 2013, Patel et al. 2018). Since ATP is a universal P2 receptor agonist it is not possible to determine which P2 receptor subtype might be mediating this effect. Furthermore, it is likely that some of these inhibitory effects are mediated via purinergic independent mechanisms, as is seen in both osteoblasts and VSMCs (Patel et al. 2018).

It has also been shown that ATP, acting via the P2Y_2_ receptor, is capable of inducing its own release from osteocytes, osteoblasts and leukocytes (Kringelbach et al. 2014; De Ita et al. 2016; Orriss et al. 2017). Consistent with these earlier studies, calcifying tendon-derived cells cultured with ATP displayed an increased level of ATP release. Interestingly, this effect was not observed in the non-calcifying cells. This suggests that the changes that occur in these cells during calcification make them more responsive to extracellular ATP. The underlying reason for increased ATP release in these conditions is unclear, however, it could represent a feedback mechanism whereby the cells are responding to the calcification and trying to inhibit it.

When considering the response of tendon cells to calcifying media at the gene level, the upregulation of osteogenic markers *SPP1* and *BGLAP* in calcifying cells indicate that these cells are taking on characteristics more typically associated with bone-forming osteoblasts. This suggests that the development of calcification is causing phenotypic changes in the tendon-derived cells, something that is also observed during the development of vascular calcification (Patel et al. 2019). Indeed, gene expression and protein-level analysis of biopsies from calcifying and non-calcifying regions of the human rotator cuff have shown an increase in SPP1/osteopontin within calcifying regions of the tendon (Takeuchi et al. 2001; Oliva et al. 2011; Grases et al. 2015; Cho et al. 2020). By contrast, induction of calcification with 10 mM BGP in rotator cuff derived cells did not regulate expression of *SPP1* or *BGLAP* (Darrieutort-Laffite et al. 2019). These results indicate that the model of calcification developed in the current study may be more representative of in vivo calcification.

While exogenous ATP had no effect on gene expression under non-calcifying conditions, it ameliorated the changes in gene expression seen in calcifying cells compared to non-calcifying cells. This indicates that the ATP-mediated inhibition of calcification is preventing the phenotypic changes in TDCs that appear to occur during the development of calcification. Interestingly, an upregulation of the P2X7 receptor gene was observed in calcifying cells treated with ATP compared to non-treated calcifying cells. Previous work has shown that the P2X7 receptor plays a role in mediating ATP release from a range of cell types including bone cells, astrocytes and skin fibroblasts (Suadicani et al. 2006; Brandao-Burch et al. 2012; Flores-Muñoz et al. 2021). Therefore, this increase in P2X7 receptor expression could explain the increase in ATP release observed in tendon-derived cells. The P2X7 receptor has also been suggested to mediate some of the inhibitory effects of ATP on bone mineralisation (Orriss et al. 2012). Further work is clearly required to determine the role of the P2X7 receptor in the context of tendon calcification.

In conclusion, we have developed a novel in vitro model of tendon calcification, and used this model to demonstrate that extracellular ATP is able to inhibit the formation of calcification in tendon-derived cells. Future work should be conducted to establish the specific pathways driving tendon calcification and if these are similar in cells from normal and diseased tendons.

### Supplementary Information

Below is the link to the electronic supplementary material.

Supplementary file1 (TIF 460 KB)

Supplementary file2 (DOCX 13 KB)

## Data Availability

The datasets generated during the current study are available from the corresponding author on reasonable request.
