# Supplementary figures and images for: Pathological calcification in canine tendon-derived cells is modulated by extracellular ATP
Source: Vet Res Commun. 2024 Feb 21;48(3):1533–43. doi: 10.1007/s11259-024-10331-1 (PMC11147865; doi:10.1007/s11259-024-10331-1)

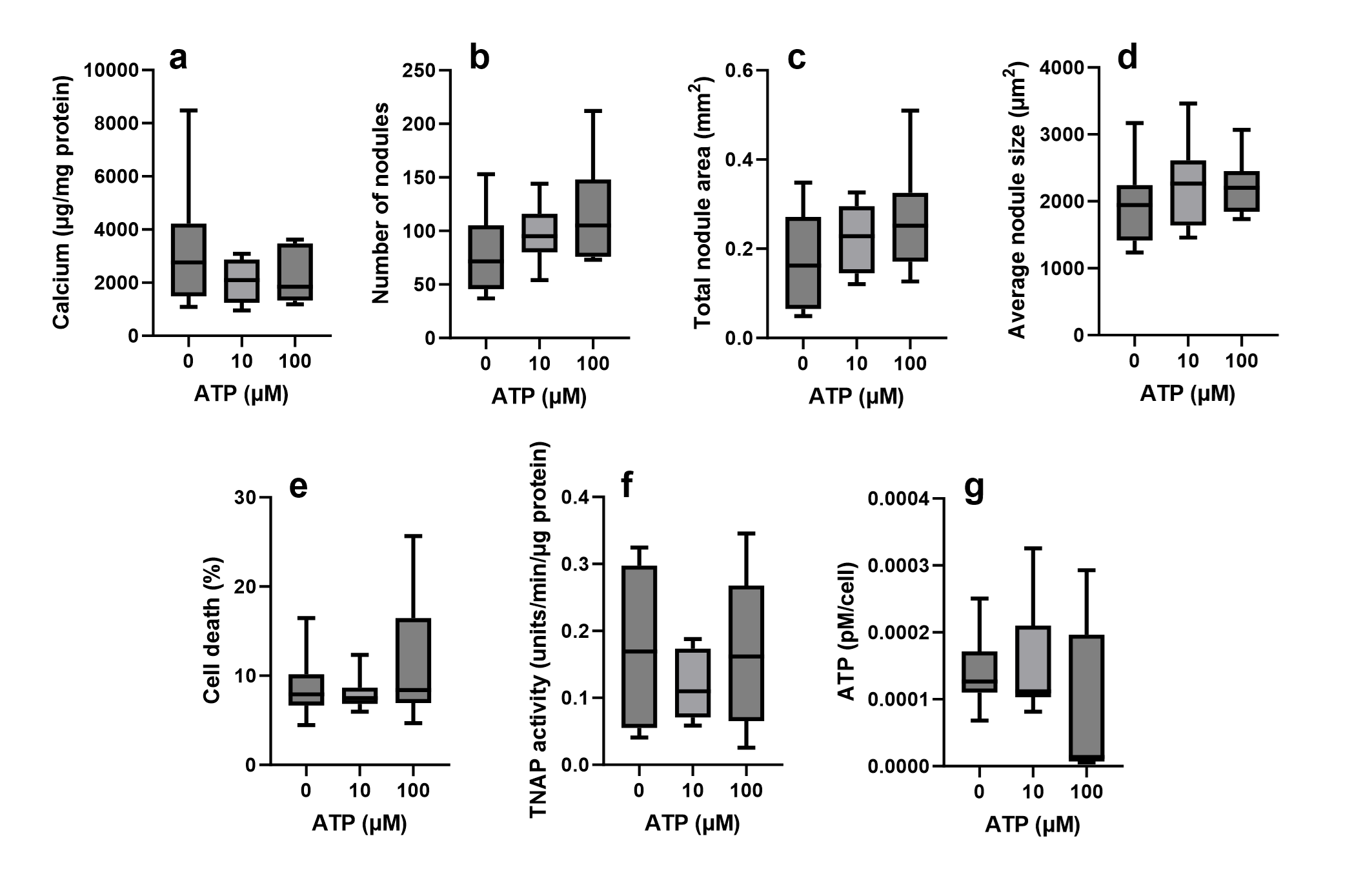

Supplement: Supplementary file 1 — Supplementary file1 (TIF 460 KB) [file 11259_2024_10331_MOESM1_ESM.tif]
