# Supplementary material for: Pathological calcification in canine tendon-derived cells is modulated by extracellular ATP
Source: Vet Res Commun. 2024 Feb 21;48(3):1533–43. doi: 10.1007/s11259-024-10331-1 (PMC11147865; doi:10.1007/s11259-024-10331-1)
Supplement: Supplementary file 2 — Supplementary file2 (DOCX 13 KB) [file 11259_2024_10331_MOESM2_ESM.docx]

**Supplementary table 1** – List of genes included in custom array

| **Gene Name**   \| *ACVR1* \| \| --- \| \| *AHSG* \| \| *ALPL* \| \| *BGLAP* \| \| *BGN* \| \| *BMP2* \| \| *BMP4* \| \| *BMP7* \| \| *COL1A1* \| \| *COL1A2* \| \| *COL2A1* \| \| *COL3A1* \| \| *COL4A1* \| \| *COL12A1* \| \| *COL14A1* \| \| *COMP* \| \| *DCN* \| \| *ENPP1* \| \| *FGF1* \| \| *FGF2* \| \| *FGFR1* \| \| *FGFR2* \| \| *FMOD* \| \| *IGFBP1* \| \| *P2RX4* \| \| *P2RX7* \| \| *RUNX2* \| \| *SOST* \| \| *SP7* \| \| *SPP1* \| \| *TGFB1* \| \| *TGFB2* \| \| *TGFBR1* \| \| *TGFBR2* \| \| *TNC* \| \| *TNMD* \| |
| --- | --- | --- | --- | --- | --- | --- | --- | --- | --- | --- | --- | --- | --- | --- | --- | --- | --- | --- | --- | --- | --- | --- | --- | --- | --- | --- | --- | --- | --- | --- | --- | --- | --- | --- | --- | --- |
